# Early Postnatal In Vivo Gliogenesis From Nestin-Lineage Progenitors Requires Cdk5

**DOI:** 10.1371/journal.pone.0072819

**Published:** 2013-08-26

**Authors:** David Petrik, Sanghee Yun, Sarah E. Latchney, Sohail Kamrudin, Junie A. LeBlanc, James A. Bibb, Amelia J. Eisch

**Affiliations:** 1 Department of Psychiatry, The University of Texas Southwestern Medical Center, Dallas, Texas, United States of America; 2 Department of Neurology, The University of Texas Southwestern Medical Center, Dallas, Texas, United States of America; University of Washington, United States of America

## Abstract

The early postnatal period is a unique time of brain development, as diminishing amounts of neurogenesis coexist with waves of gliogenesis. Understanding the molecular regulation of early postnatal gliogenesis may provide clues to normal and pathological embryonic brain ontogeny, particularly in regards to the development of astrocytes and oligodendrocytes. Cyclin dependent kinase 5 (Cdk5) contributes to neuronal migration and cell cycle control during embryogenesis, and to the differentiation of neurons and oligodendrocytes during adulthood. However, Cdk5’s function in the postnatal period and within discrete progenitor lineages is unknown. Therefore, we selectively removed Cdk5 from nestin-expressing cells and their progeny by giving transgenic mice (nestin-CreERT2/R26R-YFP/CDK5^flox/flox^ [iCdk5] and nestin-CreERT2/R26R-YFP/CDK5^wt/wt^ [WT]) tamoxifen during postnatal (P) days P2-P 4 or P7-P 9, and quantified and phenotyped recombined (YFP+) cells at P14 and P21. When Cdk5 gene deletion was induced in nestin-expressing cells and their progeny during the wave of cortical and hippocampal gliogenesis (P2-P4), significantly fewer YFP+ cells were evident in the cortex, corpus callosum, and hippocampus. Phenotypic analysis revealed the cortical decrease was due to fewer YFP+ astrocytes and oligodendrocytes, with a slightly earlier influence seen in oligodendrocytes vs. astrocytes. This effect on cortical gliogenesis was accompanied by a decrease in YFP+ proliferative cells, but not increased cell death. The role of Cdk5 in gliogenesis appeared specific to the early postnatal period, as induction of recombination at a later postnatal period (P7-P9) resulted in no change YFP+ cell number in the cortex or hippocampus. Thus, glial cells that originate from nestin-expressing cells and their progeny require Cdk5 for proper development during the early postnatal period.

## Introduction

Dysregulation of oligodendrocytes and astrocytes is implicated in a growing number of neurological disorders [1–3]. Understanding the molecular underpinnings that regulate glia during both normal and pathological development will be important in developing future avenues for early diagnosis and treatment of these disorders. While much is known about the molecular control of glia during embryogenesis [4], less is known about the molecular control of glia during early postnatal development. This is in part due to the challenges of dissecting such molecular mechanisms during a period when oligodendrocytes and astrocytes continue to proliferate and migrate well after the generation and migration of most neurons are complete [5].

Some general aspects of glia development during the early postnatal period have been established. For example, postnatal astrocytes and oligodendrocytes emerge from a variety of precursors, including radial glial cells, glioblasts in the subventricular zone, oligodendrocytic-specific progenitors, and – in some brain regions like the cerebral cortex – local sources [6–10]. Oligodendrocytes populate the brain in several waves, with the first two waves starting at embryonic day (E) 12.5 and E16.5, respectively, and the last cell wave starting at birth (P0) and lasting through the first few postnatal weeks (until ~P14) [6,11,12]. In contrast to oligodendrogenesis, astrogliogenesis is less understood, likely due to a lack of cell markers specific to astrocytic precursors [4]. However, astrocytic precursors appear to emerge from radial glia cells that enter different brain regions also during the early postnatal period (e.g. P0-P7) [4,13–15]. Given that oligodendrocyte and astrocyte precursors express the nestin early in development [6,15,16] and that several lines of nestin-inducible fate-tracking mice exist [17], it is notable that to-date no studies have specifically targeted the early postnatal period to assess glial progeny from nestin-expressing cells. In addition, few studies have used inducible gene deletion approaches to assess the molecular basis of early postnatal gliogenesis.

One of the most important regulators of neuronal development is cyclin-dependent kinase 5 (Cdk5). During embryogenesis and early life, this kinase regulates cell cycle reentry and neuronal migration [18–20]. During adulthood, Cdk5 is critical for neuronal differentiation and proper dendritic morphology in the hippocampal neurogenic niche, the subgranular zone [21,22]. In addition to its role in neuronal development, correlative and *in vitro* evidence suggests Cdk5 may also be involved in glial development. For example, Cdk5 is expressed in both oligodendrocytes and astrocytes [23], phosphorylates the astrocytic protein GFAP *in vitro*, and appears to be important for the astrocyte cytoskeletal dynamics [24,25]. However, direct evidence for the role of Cdk5 in astrocytes and oligodendrocytes is limited. For example, nothing is known about the role of Cdk5 in normal astrogenesis *in vivo* during early life. Cdk5’s role in oligodendrocytes is slightly better understood; *in vitro* it appears critical for their migration and their differentiation [26–28], and *in vivo* it is critical for their differentiation [29]. As with astrocytes, relatively little is understood about the specific role of Cdk5 in early postnatal oligodendrogenesis.

To investigate the role of Cdk5 in early postnatal brain development and gliogenesis in particular, we employed our nestin-CreERT2 mice [30,31] and Cre recombinase-sensitive Cdk5 mice [32] to allow inducible deletion of Cdk5 from nestin-expressing cells and their progeny during the postnatal development in mice. Cdk5 is expressed in oligodendrocytes and astrocytes [23], nestin-expressing progenitors can generate oligodendrocytes, astrocytes, stem cells, and neurons [6,15], and Cdk5 is critical for the proliferation and survival during development [33]. Therefore, we hypothesized that tamoxifen(Tam)-induced deletion of Cdk5 in nestin-expressing cells and their progeny during the early postnatal period would diminish glial production from nestin-expressing cells. In keeping with our hypothesis, inducible deletion of Cdk5 from nestin-expressing cells led to fewer recombined astrocytes and oligodendrocytes in the cortex, hippocampus, and corpus callosum. The role of Cdk5 in gliogenesis was restricted to early life, as inducible deletion after the first postnatal week had no effect on recombined cells. These data suggest glial cells originating from nestin-expressing cells and their progeny require Cdk5 for proper development during the early postnatal period.

## Methods

### Animals and Ethics Statement

All experiments were approved by the Institutional Animal Use and Care Committee at UT Southwestern (UTSW) Medical Center (Permit Number: APN 0960-07-02-1). Nestin-CreERT2 and CDK5^flox/flox^ mice – both previously published and characterized [31,32] – were bred at UTSW; Nestin-CreERT2 mice are also available via Jackson Laboratories (strain #016261). R26R-YFP mice – a widely-used Cre recombinase reporter line (strain #006148 [34]) – were received from Jackson Laboratories and bred at UTSW. Mice were genotyped as previously described [21]. All mice were group-housed in a UTSW vivarium accredited by the Association for Assessment and Accreditation of Laboratory Animal Care (AAALAC), and were kept on a 12-hour (hrs) light/dark cycle with *ad libitum* access to food and water. Mice were first bred to generate fathers homozygous for nestin-CreERT2 and hemizygotic for Cdk5 flanked by loxP (floxed) sites (Cdk5^flox/wt^), and mothers homozygous for the reporter transgene R26R-YFP and hemizygotic for Cdk5 (Cdk5^flox/wt^). Pairing of these parents resulted in expected litters according to Mendelian ratios, including 25% wildtype (WT; nestin-CreERT2/R26R-YFP/Cdk5^wt/wt^) and 25% nestin-inducible knockout mice (iCdk5; nestin-CreERT2/R26R-YFP/Cdk5^flox/flox^). Thus, WT and iCdk5 littermates were used for quantitative comparison at a single timepoint analysis (e.g. P14 or P21), with different litters used for different timepoints. As the primary comparisons for this manuscript were between WT and iCdk5 mice, only qualitative analyses were performed between timepoints (e.g. P14 vs. P21). While most experiments in this study used WT and iCdk5 littermates, one pilot experiment used nestin-CreERT2/R26R-YFP (nestin-YFP) littermates to assess effectiveness of different Tam administration paradigms (Fig. S1). For all studies presented here, pups were sacrificed via decapitation at P14 or P21, a procedure approved by the UTSW Institutional Animal Care and Use Committee.

### Drug Administration

Mice were given Tam (Sigma; 30 mg/ml of 10% ethanol and 90% sunflower oil) in 2 different paradigms [35,36]. For pilot studies (Figure S1), Tam (150 mg/kg) was given only to lactating mothers (termed “Tam to mother”, or “TOM”) via 1 daily intraperitoneal (i.p.) injection for 3 days when the pups were P2-P4. For all other experiments, Tam was given to mothers and to pups (“TOM-TOP”) of WT and iCdk5 mice, with mothers receiving the daily i.p. injections and pups receiving daily oral (p.o. via P20 pipette) Tam from either P2-P4 or P7-P9.

### Immunohistochemistry (IHC)

Brains were extracted, immersion-fixed in 4% paraformaldehyde, and processed for slide-mounted and free-floating IHC as previously described [31,37]. A freezing microtome was used to collect 30 µm coronal sections throughout the entire hippocampus in serial sets of 8 (for P14) or 9 (for P21) for IHC to visualize immunoreactive(+) cells. The following primary antibodies were used: chicken polyclonal anti-green fluorescent protein (GFP; 1:3000; Aves Labs, Tigard, OR) [37]; mouse polyclonal anti-glial fibrillary acidic protein (GFAP; 1:400; Millipore, Billerica, MA) [37]; mouse polyclonal anti-oligodendrocyte (RIP; 1:200; Developmental Studies Hybridoma Bank, DSHB, Iowa City, IA) [38,39]; mouse polyclonal IgM anti-4D4 (1:40; DSHB, Iowa City, IA) [40]; rabbit polyclonal anti-S100b (1:400; SWANT, Marly, Switzerland) [37]; rabbit polyclonal anti-Ki67 (1:200; Thermo Scientific, Rockford, IL) [37]; or rabbit polyclonal anti-activated caspase 3 (AC3; 1:500; Cell Signaling Technologies, Danvers, MA) [37]. All secondary antibodies (1:200) were from Jackson ImmunoResearch, West Grove, PA, with exception for goat anti-mouse IgM mu chain conjugated to DyLight 488 (1:200; AbCam, Cambridge, MA).

For stereologic quantification of YFP+ or AC3+ cells and qualitative and phenotypic analysis of YFP+ cells [31,37], slide-mounted sections were pre-treated in citric acid and then incubated with primary antibody against GFP overnight, with subsequent 60 min incubations in biotin-conjugated secondary antibody (1:250) and avidin-biotin complex (1:50; Vector Laboratories, Burlingame, CA). Visualization was performed by incubation with Tyramide-Plus signal amplification (1:50; PerkinElmer, Boston, MA) for YFP+ cells, and DAB/metal concentrate (10x; Thermo Scientific) for AC3+ cells. For double- or triple-labeling and subsequent phenotypic analysis, tissue was incubated with 2 primary antibodies (YFP and either 4D4 or S100b) or 3 primary antibodies (YFP, Ki67 and either GFAP or RIP) on slide-mounted sections overnight. For brightfield microscopy, slides were counterstained with Nuclear Fast Red (Vector). For epifluorescent microscopy, slides were counterstained with DAPI (1:5000; Roche). All slides were dehydrated and coverslipped using DPX (Steinheim, Germany).

### Qualitative analysis, cell quantification, and phenotypic analysis

To assess the efficacy of TOM or TOM-TOP in driving Tam-induced Cre-mediated recombination in the brain, YFP-stained brain sections from nestin-YFP mice were examined at low and high magnification for presence or absence of YFP+ cells in any region of the brain. For these pilot studies of TOM vs. TOM-TOP efficacy, 3 litters were examined, with an average of 8 pups/litter.

To quantify YFP+ and AC3+ cells, stained brain sections from WT and iCdk5 TOM-TOP mice were examined via an observer blind to treatment group using published stereological principles [41]. YFP+ cells were quantified in cortex, corpus callosum (cc), and hippocampus, specifically in the stratum oriens (Or), and stratum radiatum (Rad). AC3+ cells were quantified in cortex. For both the cortex and corpus callosum, the anterior–posterior boundary was defined by sections in which the hippocampus was evident, plus “bookended” sections (e.g. one section prior and one section subsequent to the hippocampus). For the cortex, the medial-lateral boundary was defined as the medial tip of the midsagittal sulcus to the ventral lateral edge of the cortex. Therefore, cortical analysis included the majority of the retrosplenial agranular, visual, auditory, and entorhinal cortices and the posterior aspects of the sensory, motor, and piriform cortices, but excluded medial structures like the cingulate and retrosplenial granular cortices and subcortical structures like the amygdala [42]. Cortical analysis also included layers I-VI. For the corpus callosum, the medial-lateral boundary was defined as the midsagittal or medial appearance to the ventral lateral boundary. While the cingulum was considered for corpus callosum analysis, regions of the corpus callosum that were ventral and lateral to the callosal bifurcation (e.g. near amygdala) were excluded. Data are presented as total cells per region analyzed (mean±standard error of the mean [s.e.m.]). For these studies – which comprise most figures in this study – each time point and genotype represents a minimum of 4-5 mice.

After stereological cell quantification, a subset of animals (with average YFP+ cell counts, 3-4 mice per timepoint and genotype) was selected for additional phenotypic analysis of double-labeled cells. Phenotypic analysis was performed using laser scanning confocal microscopy as previously described [43,44] to assess the proportion of YFP+ cells in the cortex were that were RIP+YFP+, GFAP+YFP+, S100b+YFP+, 4D4+YFP+, Ki67+YFP+, Ki67+GFAP+YFP+, and Ki67+RIP+YFP. The glial antibodies (GFAP, RIP, S100b, and 4D4) marked cell bodies, many which had characteristic glial processes emerging from the soma. Colocalization, however, was quantified only if the YFP+ and glial antibody signals colocalized to the soma under three-dimensional/orthogonal confocal Z-scan analysis. Phenotypic proportions were then multiplied by total number of YFP+ cells (from stereology), and the resulting data on the total number of double+ (or triple+) cells per brain region are reported (mean±s.e.m.).

### Volume quantification

Volume of the cortex and hippocampus was quantified using the Cavalieri Probe within Stereo Investigator (MBF Bioscience, MicroBrightField, Inc., Williston, Vermont) as previously published [45,46]. For P14 and P21 brains, every 8^th^ (P14) or 9^th^ section (P21) from -0.94 to -3.64 mm from Bregma was analyzed to assess the size of the cortex and hippocampus. The anatomical boundaries that were used to quantify YFP+ cells in the cortex and hippocampus were also applied for Cavalieri volume measurements. The region of interest was viewed and volume was determined using an Olympus BX51 microscope with a MicroFIRE A/R camera (Optronics, Goleta, California) and 10x, NA 0.30 lens. Area sizes were determined using the area measurement tool (based on the Cavalieri estimator) in which an automated grid of test points was superimposed upon the region of interest. Area was estimated from the total number of points that fell within the respective field. To obtain the volume, the sum of the areas measured was multiplied by the sampling fraction (8 for P14 and 9 for P21) and the section thickness (30µm). The Gunderson coefficient of variance for each mouse quantified was always less than 10%. Data are reported as the total estimated volume (mm^3^) of the cortex and hippocampus per brain and are presented as mean±s.e.m.

### Statistical analysis

The experiments were designed to specifically assess the role of Cdk5 in postnatal development. Therefore, the primary comparisons of interest were between WT and iCdk5 mice at each timepoint. Therefore, unless specified otherwise, data were compared with an unpaired Student’s t-test between WT and iCdk5 mice at a given timepoint (e.g. Tam P2-P4, kill P14). Significance value was set at p<0.05 and indicated as an asterisk. Significance value at p<0.07 is indicated by a pound sign.

## Results

### Administration of Tam to mothers and to pups is an effective approach to induce genetic deletion in nestin-lineage cells

To study the role of Cdk5 in early postnatal development while leaving embryonic development intact, we first established that we could initiate recombination in the early postnatal brain. We compared Tam administration to nestin-YFP mothers (TOM) to Tam administration to nestin-YFP mothers and their P2-P4 pups (TOM-TOP; Figure S1). Our readout for these initial studies was the presence of YFP+ cells in the brain of nestin-YFP mice, a sign of Tam-induced Cre-mediated recombination in nestin-expressing cells. The qualitative analysis revealed that TOM (given P2-P4, kill P21) resulted in YFP+ cells in only 60% of pups in a litter with YFP+ brain cells (Figure S1A), while TOM-TOP P2-P4 resulted in 100% of pups in a litter. Thus, all other experiments presented here utilized the TOM-TOP approach.

With the TOM-TOP paradigm, both Cdk5 WT (nestin-CreERT2/R26R-YFP/Cdk5^wt/wt^) and iCdk5 mice (nestin-CreERT2/R26R-YFP/Cdk5^flox/flox^) had normal weight gain over time with Tam P2-P4 (Figure S1B) or Tam P7-P9 (Figure S1C). Both mice had normal body and overall brain development, and displayed normal general activity until time of kill (data not shown). Thus, unlike constitutive deletion of Cdk5 [47], deletion of Cdk5 from nestin-expressing cells in the early postnatal period has no overt effect on indices of general health. Given the efficacy and safety of the TOM-TOP approach, all subsequent experiments used TOM-TOP, indicated as “Tam P2-P4” or “Tam P7-P9”.

### Inducible deletion of Cdk5 in nestin-lineage cells (Tam P2-P4) results in fewer YFP+ glial cells in cortex

**Figure 1:**
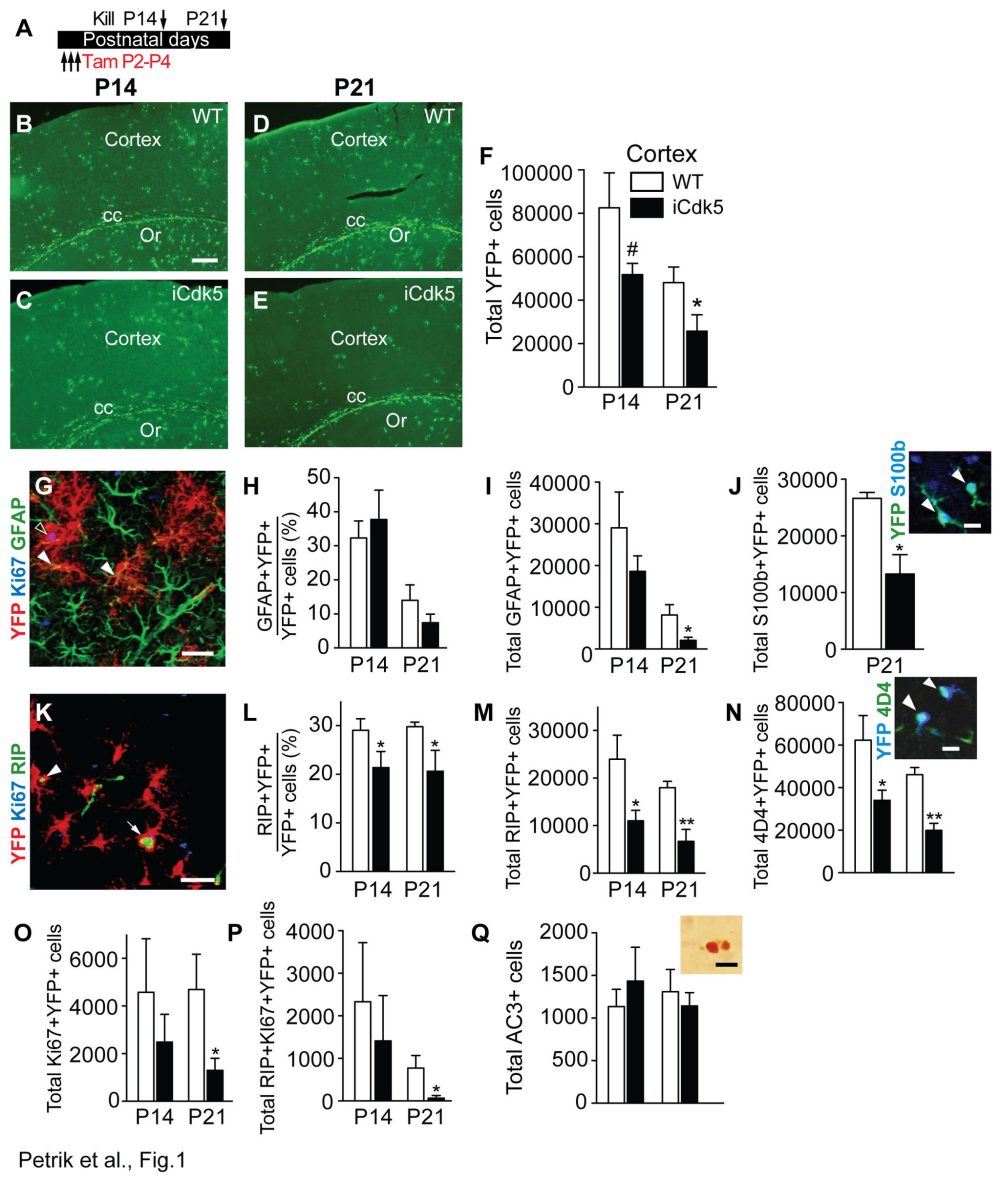
Inducible deletion of Cdk5 in nestin-lineage cells (Tam P2-P4) results in fewer YFP+ glial cells in cortex. (**A**) Paradigm of Tam administration. Representative images for brain sections stained for YFP at P14 (**B**, **C**) and at P21 (**D**, **E**) show YFP+ cells in the cortex, corpus callosum (cc), and stratum oriens (Or) of the hippocampus in the WT (top) and iCdk5 littermates (bottom; scale bar, s.b.=100 µm). (**F**) Stereological quantification of YFP+ cells in the cortex. (**G**) Representative microphotograph of cortex shows YFP+ cells (red) overlapping with GFAP (green; closed arrowheads) or with GFAP and Ki67 (blue; open arrowhead; s.b.=20 µm). (**H**) Percent of YFP+ cortical cells expressing both GFAP and YFP. (**I**) Total number of GFAP+YFP+ cortical cells. (**J**) Total number of S100b+YFP+ cortical cells at P21. Inset shows double-positive cells (arrowheads; s.b.=10 µm). (**K**) Representative image of cortex shows YFP+ cells (red) overlapping with RIP alone (green; arrowhead), or with RIP and Ki67 (arrow; s.b.=20 µm). (**L**) Percent of YFP+ cortical cells expressing both RIP and YFP. (**M**) Total number of RIP+YFP+ cortical cells. (**N**) Total number of 4D4+YFP+ cortical cells at P21. Inset shows double-positive cells (arrowheads; s.b.=10 µm). (**O**) Total number of Ki67+YFP+ cortical cells. (**P**) Total number of RIP+Ki67+YFP+ cortical cells. (**Q**) Total number of AC3+ cortical cells (inset shows two AC3+ cells; s.b.=10 µm).

Having established the general efficacy and safety of giving Tam via TOM-TOP, we began our cellular investigation into the role of Cdk5 in nestin-lineage cells during postnatal development. We first performed a qualitative examination of YFP+ cell distribution in the brains of WT and iCdk5 mice given Tam P2-P4 and killed at P14 or P21, with an emphasis on gross distribution in WT brains (Figure 1A–E). At both P14 and P21 there were YFP+ cells in the gliogenic regions of the brain – cortex, hippocampus, and corpus callosum. In addition, there were relatively fewer cells evident at P21 compared to P14, although our experimental design did not allow statistical analysis of this impression. This qualitative distribution of YFP+ cells fits with the known contribution of nestin-lineage cells to development of these brain regions during the postnatal period.

Given that the cortex completes cytogenesis and migration prior to the hippocampus [48], the cortex was the first focal point of our analysis of YFP+ cell number and phenotype. We hypothesized that inducible deletion of Cdk5 from nestin-lineage cells would result in fewer YFP+ cortical cells, as predicted from the role of Cdk5 during earlier and later periods of development in the cortex and other brain regions [20]. In keeping with our hypothesis, quantitative analysis showed that inducible deletion of Cdk5 from nestin-lineage cells (Tam P2-P4) resulted in fewer YFP+ cortical cells than in WT littermates when examined 2-3 weeks later (Figure 1F). There was a strong trend towards fewer YFP+ cells in iCdk5 vs. WT littermates at P14 (p=0.06), and significantly fewer YFP+ cells in iCdk5 vs. WT littermates at P21 (p<0.05). These data show that inducible deletion of Cdk5 from nestin-lineage cells during a period of active cortical development and gliogenesis decreases the number of YFP+ progeny in the cortex 2-3 weeks later.

Given the ongoing gliogenesis in the postnatal cortex [6,11,15], we next hypothesized inducible deletion of Cdk5 from nestin-expressing cells would result in fewer YFP+ cortical glial cells. Indeed, the YFP+ cortical cells presented a variety of morphologies, including oligodendrocytic and astrocytic cells but rarely neurons (Figure 1B–E), in keeping with data that nestin-lineage cells generate to glia in the developing cortex. Phenotypic analysis of YFP+ cortical cells in P14 WT mice given Tam P2-P4 revealed that about one-third of YFP+ cells expressed a marker of astrocytes, GFAP+ (Figure 1G–I) [49,50], and one-third expressed a marker of oligodendrocytes, RIP (Figure 1K–M) [51,52]. In P21 WT mice given Tam P2-P4, the GFAP+YFP+ proportion was lower than the RIP+YFP+ proportion (Figure 1H vs. 1L). By multiplying these proportions with the absolute number of YFP+ cell number from the stereologic assessment [37], a picture emerged of the dynamic and ongoing astrogenesis and oligodendrogenesis in the WT mouse cortex (Figure 1I vs. 1M). At P14, there were roughly equal numbers of GFAP+YFP+ astrocytes and RIP+YFP+ oligodendocytes. At P21, there were fewer GFAP+YFP+ astrocytes than RIP+YFP+ oligodendrocytes. These results in WT mice confirm that – as suggested using other inducible mice and other peri- and postnatal time points for induction – nestin-expressing cells give rise to oligodendrocytes and astrocytes during the first postnatal week in mice.

Having established the distribution of the glial phenotypes of nestin-lineage cortical cells in WT mice, we next examined the distribution of glial phenotypes after inducible deletion of Cdk5 from nestin-lineage cells (Tam P2-P4). Proportionally, there was a lower percentage of RIP+YFP+ cells in iCdk5 vs. WT mice at both P14 and P21 (Figure 1L), with no similar shift in GFAP+YFP+ cells (Figure 1H) or YFP+ cells positive for another astrocytic marker, a calcium-binding protein S100b (proportional data not shown) [53]. Multiplication of these proportional data by the total respective cell numbers revealed fewer GFAP+YFP+ at P21 (Figure 1I; along with a non-significant trend at P14) and fewer S100b+YFP+ cells at P21 (Figure 1J) in iCdk5 vs. WT mice at P21, while there were fewer RIP+YFP+ cells in iCdk5 vs. WT mice at both P14 and P21 (Figure 1M). This reduction in number of mature astrocytes and oligodendrocytes in iCdk5 vs. WT was mirrored by reduction in number of YFP+ cells labeled for a marker of oligodendrocyte-astrocyte precursors, 4D4 (Figure 1N) [40,54].

One possible explanation for reduced gliogenesis in iCdk5 mice is reduced cortical volume. However, the total volume of the cortex did not differ between WT and iCdk5 at P14 (mean[mm^3^] ±s.e.m WT: 35.3±2.2; iCdk5: 35.8±3.5; p>0.05) or at P21 (WT: 37.7±1.9; iCdk5: 41.9±1.6; p>0.05). Moreover, when the total cortical volume was factored into the total number of GFAP+YFP+ astrocytes and RIP+YFP+ oligodendrocytes, there was an identical reduction in astrogenesis and oligodendrogenesis as seen in Figure 1I and 1M (data not shown). Thus, inducible deletion of Cdk5 in nestin-lineage cells from P2-P4 results in fewer YFP+ astrocytes and oligodendrocytes in the cortex without significantly changing cortical volume when examined 2-3 weeks later.

### Inducible deletion of Cdk5 in nestin-lineage cells (Tam P2-P4) results in fewer YFP+ proliferative cells in cortex

The reduction in cortical YFP+ astrocytes and oligodendrocytes in iCdk5 vs. WT mice could be due to many underlying mechanisms, including impaired proliferation or increased cell death. To address the possibility that inducible deletion of Cdk5 in nestin-lineage cells impaired proliferation in the cortex (Tam P2-P4), we examined the proportion of YFP+ cells that expressed Ki67, an endogenous proliferation marker of neuronal and glial precursors [55–57], and then calculated the number of Ki67+YFP+ cortical cells (Figure 1O). At P14, there was an apparent trend towards fewer Ki67+YFP+ cortical cells in iCdk5 vs. WT mice but this was not significant (p>0.6). However, at P21 iCdk5 mice presented 70% fewer Ki67+YFP+ cells than WT mice (Figure 1O). Moreover, since total cortical volume was not significantly different between iCdk5 and WT mice at P14 or P21 (see above), the proportional reduction of Ki67+YFP+ cortical cells in iCdk5 vs. WT mice with respect to total cortical volume remained identical (data not shown). To phenotype these Ki67+YFP+ cells, we examined their colocalization with GFAP or RIP. We observed almost no GFAP+Ki67+YFP+ cells in either WT or iCDK5 mice. However, there was a greater than 90% reduction in RIP+Ki67+YFP+ cells in iCDK5 mice vs. WT at P21 (Figure 1P), with a trend towards a decrease seen at P14. To address the companion possibility – that inducible deletion of Cdk5 in nestin-lineage cells increased cell death – we examined the total number of cortical cells that expressed AC3, a marker of apoptosis [58]. However, WT and iCdk5 mice did not differ in the number of cortical AC3 cells at either P14 or P21 (Figure 1Q). Taken together, these data show that inducible deletion of Cdk5 in nestin-lineage cells via Tam P2-P4 decreased YFP+ astrocytes, oligodendrocytes, and proliferative cells in the cortex.

### Inducible deletion of Cdk5 in nestin-lineage cells (Tam P2-P4) also results in fewer YFP+ cells in other gliogenic regions

**Figure 2:**
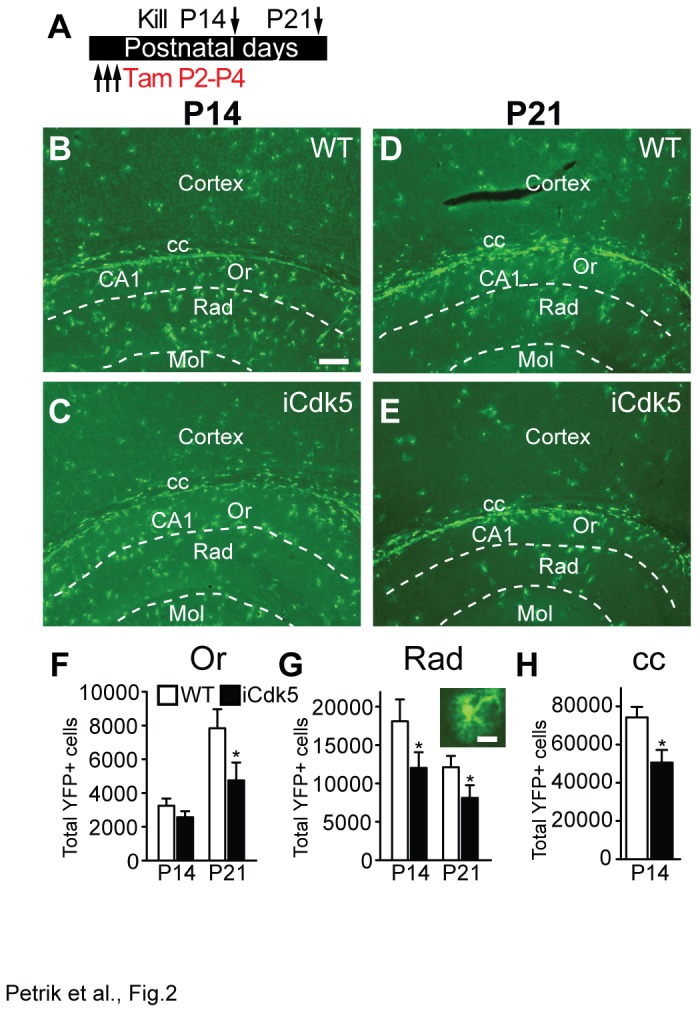
Inducible deletion of Cdk5 in nestin-lineage cells results in fewer YFP+ cells in the hippocampus and corpus callosum. (**A**) Paradigm of Tam administration. Representative images of the brain at P14 (**B**, **C**) and P21 (**D**, **E**) at P14 in WT (top) and iCdk5 littermates (bottom) highlighting cortex, corpus callosum (cc), and hippocampus with stratum oriens (Or), CA1, stratum radiatum (Rad) and molecular layer (Mol, s.b.=100 µm). Total number of YFP+ cells in Or (**F**), Rad (**G**; inset shows typical YFP+ Rad cell at P14, s.b.=10 µm), and cc (**H**).

Having established that loss of Cdk5 from nestin-lineage cells via Tam from P2-P4 led to fewer cortical YFP+ astrocytes and oligodendrocytes, we turned our attention to 2 other regions of ongoing gliogenesis: the hippocampus and corpus callosum (Figure 2A–E). The reduction in the number of YFP+ glial cells in the cortex was mirrored in the hippocampus, where 2 layers in the Cornu Ammon’s (CA1) region of the hippocampus had fewer YFP+ cells. Both the stratum oriens (Or; Figure 2F) and the stratum radiatum (Rad; Figure 2G) had fewer YFP+ cells in iCdk5 mice relative to WT littermates. The YFP+ cells in the CA1 region presented an astrocytic-like morphology (Figure 2G), similar to the YFP+ cortical astrocytes. Similar to the cortex, the reduction in YFP+ cells in the hippocampus of iCdk5 mice was not due to smaller hippocampal volume, as the total hippocampal volume did not differ between WT and iCdk5 mice at P14 (WT: 13.3±0.8; iCdk5: 13.2±1.2; p>0.05) or P21 (WT: 13.7±1.6; iCdk5: 14.7±1.8; p>0.05). In addition there were fewer YFP+ cells in the corpus callosum of iCdk5 vs. WT mice at P14 (Figure 2H), but no significant difference AC3+ cells in the corpus callosum (WT: 598.9±89.4; p>0.05; iCdk5: 512±111.0; p>0.05). Taken together, these results show inducible deletion of Cdk5 from nestin-lineage cells via Tam P2-P4 results in fewer YFP+ cells in the cortex, corpus callosum, stratum oriens, and stratum radiatum regions of CA1 when examined 2-3 weeks later.

### In contrast to the effects via Tam P2-P4, inducible deletion of Cdk5 in nestin-lineage cells via Tam P7-P9 does not change YFP+ cell number in hippocampal or cortex

**Figure 3:**
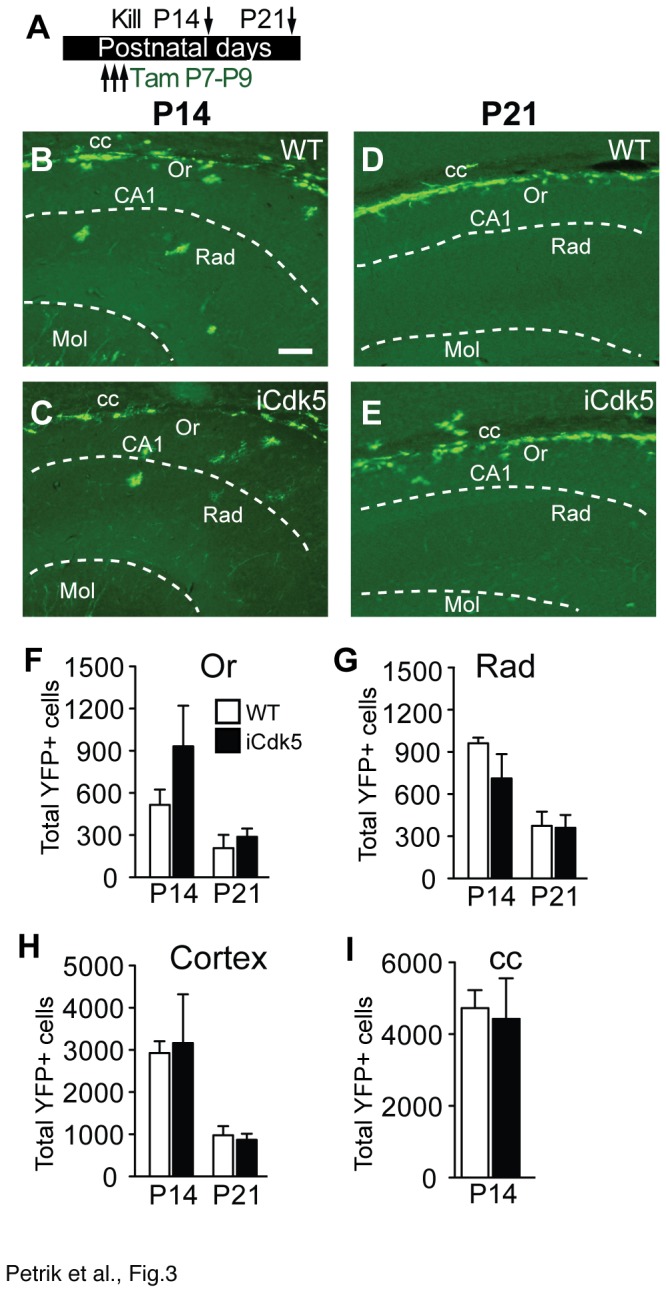
Inducible deletion of Cdk5 in nestin-lineage cells via Tam P7-P9 does not change YFP+ cell number in hippocampus or cortex. (**A**) Paradigm for Tam administration. Representative images of YFP-stained hippocampi from P14 (**B**, **C**) and P21 (**D**, **E**) show YFP+ cells in corpus callosum (cc), stratum oriens (Or) and *radiatum* (Rad) in WT (top) and iCdk5 littermates (bottom; scale bar, s.b.=100 µm). Stereological quantification of YFP+ cells in the Or (**F**), Rad (**G**), cortex (**H**) and cc (**I**).

Since inducible deletion of Cdk5 from nestin-lineage cells via Tam P2-P4 influenced YFP+ cells in ongoing regions of gliogenesis, we hypothesized deletion of Cdk5 from nestin-lineage cells after cessation of most gliogenesis would result in no change in YFP+ cell numbers. To test this, mice were given Tam via Tam P7-P9 and killed P14 or P21 (Figure 3A), and the hippocampus and cortex were stereologically examined for YFP+ cells (Figure 3B–E). Unlike inducible deletion of Cdk5 via Tam P2-P4, there was no difference between WT and iCdk5 mice in the number of YFP+ cells in CA layers (Or, Figure 3F; Rad, Figure 3G), although there was a non significant trend towards more YFP+ Or cells at P14. In addition, there was no difference between WT and iCdk5 mice in the number of YFP+ cells in the cortex (Figure 3H) and the corpus callosum (Figure 3I). When taken together, the Tam P2-P4 and Tam P7-P9 experiments suggest that postnatal generation of astrocytes and oligodendrocytes from nestin-lineage progenitors are largely restricted to the first week after birth.

## Discussion

While much is known about glial and neuronal development during embryogenesis [4,5,59], far less is known about the *in vivo* regulation of glia during the early postnatal period. It is not clear what molecular and genetic factors determine how astrocytes and oligodendrocytes populate the brain during the first weeks of postnatal life in mice. It is also unclear if the many key transcription factors, growth factors, and genetic regulators in embryogenesis are also important during the early postnatal period. An example of a protein having discrete, developmental-stage specific roles is Cdk5. Cdk5 was initially thought to be specific for neuronal development during embryogenesis [47], but subsequent studies revealed its role in many non-neuronal cells [33,60]. Despite recent progress in understanding non-neuronal Cdk5 functions [61–63], it is not clear if and how Cdk5 is involved in development of astrocytes and oligodendrocytes *in vivo*, especially in the early postnatal period. At least 2 obstacles prevented prior exploration of the regulation of postnatal gliogenesis by Cdk5. First, constitutive knockouts of Cdk5 or its activators, like p35, display increased perinatal mortality and severe developmental abnormalities [29,47,64], which complicates interpretation of effects specific to postnatal period. Second, until recently [21,32], there was a lack of appropriate inducible Cdk5 knockout mouse models that would allow temporal and specific deletion within a discrete cell population during early postnatal period when gliogenesis predominantly occurs.

We used an inducible knockout of Cdk5 in nestin-expressing cells and their progeny [21,31] to clarify the role of nestin-expressing cells in gliogenesis in early postnatal life, and to examine the role of Cdk5 in postnatal gliogenesis from nestin-expressing progenitors *in vivo*. As discussed below, our results confirm and extend prior work that astrocytes and oligodendrocytes are generated from nestin-expressing progenitors during the first postnatal week. Our results also show that Cdk5 in nestin-expressing progenitors are important for proper development of astrocytes and oligodendrocytes in early postnatal life.

### Nestin-expressing progenitors generate astrocytes and oligodendrocytes during first postnatal week

A large body of evidence shows that astrocytes and oligodendrocytes are generated during the early postnatal period [5]. Oligodendrocyte progenitors migrate into the cortex and other brain regions in 3 waves, with the first beginning at E12.5 [11,12] and the last wave occurring during the first postnatal week from the hippocampal subventricular region [6] that hosts nestin-expressing progenitors [15]. Astrocytic migration is less clear, likely because hallmark antigens such as GFAP or S100beta label both putative progenitors and mature astrocytes [4]. It is also unclear what role nestin-expressing progenitors have in glial development during early postnatal development. Certainly, nestin-expressing progenitors give rise to neurons and radial glial cells during embryogenesis, and to a small number of neurons in discrete regions of the adult brain [5]. However, prior to our study presented here, the contribution of nestin-lineage progenitor cells towards the generation of astrocytes and oligodendrocytes during early postnatal development was not clear.

To this end, we utilized nestin-CreERT2/R26R-YFP mice for lineage tracking of YFP+ cells derived from nestin-expressing progenitors. We show that glial generation from nestin-lineage progenitors is confined to the first postnatal week, as we observe more than 50-fold decrease in YFP+ cells in P21 cortex between earlier Tam administration at P2-P4 and later Tam administration at P7-P9. These results support prior work showing that both oligodendrocytes and astrocytes are generated from nestin-expressing cells [15,65] and in roughly equal numbers. While our data is largely confirmatory – agreeing with prior work showing that a significant source of early postnatal astrocytes and oligodendrocytes is nestin-expressing progenitor cells – they also extend prior work in important ways. For example, prior work showed continuous cortical postnatal gliogenesis during the first postnatal week by tracking implanted cells [14] or lineage-tracking in transgenic mice [11]. These tracked cells presented some challenges, in that they were exogenous [14], or were difficult to distinguish from cells generated during embryogenesis [11], and none of the prior work enabled distinction between local generation of astrocytes in the early postnatal cortex [10] from those that may migrate into the region. Therefore, a major benefit of our current approach is to demonstrate the efficacy of temporally-specific lineage-tracking, allowing specification of the generation of oligodendrocytes and astrocytes from nestin-expressing progenitors only during the first postnatal week. This sets the stage for future work that can address the specific cellular source or migration pattern of astrocytes vs. oligodendrocytes in the early postnatal period, for example, by using nestinCreERT2/R26R-YFP transgenic mice along with shorter survival times after Tam.

### Oligodendrocytes and astrocytes derived from nestin-expressing cells rely on Cdk5

In both embryonic and adult development, Cdk5 controls cell migration, differentiation, synaptic plasticity, and the cell cycle [33]. However, the *in vivo* role of Cdk5 in early stages of postnatal gliogenesis was previously unknown. Here we find that inducible deletion of Cdk5 from nestin-expressing cells during the early postnatal period ultimately results in fewer recombined astrocytes and oligodendrocytes. The decrease in recombined glial cells was seen in 3 regions undergoing gliogenesis – the cortex, CA1 hippocampal region, and corpus callosum – suggesting this is not a region-specific phenomenon. There may be, however, a cell type-specific role for Cdk5 in gliogenesis. For example, the decrease in recombined GFAP+ astrocyte number takes longer to appear relative to recombined RIP+ oligodendrocytes. Specifically, we observed only a trend towards fewer YFP+ astrocytes in iCdk5 vs. WT mice at P14, but significantly fewer YFP+ oligodendrocytes at P14. The mechanism for this cell-specific reduction may differ between the two glial cell types. For example, at P21 there is a reduction in both GFAP+ and RIP+ cell types, but in oligodendrocytes there may be impaired cell fate/maturation as well. These results also support recent findings that astrocytes can also be generated locally in the postnatal cortex [10], which could contribute to the prolonged time required to observe fewer recombined GFAP+ astrocytes in iCdk5 mice. It is also possible that Cdk5 may have distinct roles for regulating proliferative and post-mitotic astrocytes during gliogenesis. However, as additional astrocytic markers such as GLAST, GFAP, and S100b can label both post-mitotic astrocytes and astrocytic progenitors [4], it remains unclear from our study how Cdk5 deletion may differentially affect proliferative and post-mitotic astrocytes. Further complicating matters, there is limited consensus as to which cell lineage astrocytes follow during their development [66–70]. Therefore, future experiments are needed to assess in more detail the role of Cdk5 on proliferating vs. post-mitotic astrocytes.

What could be the possible molecular and cellular mechanisms underlying how Cdk5 supports early postnatal gliogenesis from nestin-expressing progenitors? Cdk5 controls key cytoskeleton elements – such as actin-related proteins [71] or GFAP [24,25] – to adjust the intracellular machinery during differentiation and maturation. Such a role for Cdk5 in differentiation was observed in neurons generated from nestin-expressing progenitors in adult hippocampus [21], and applies for differentiation of oligodendrocytes as well. Cdk5 activity increases during the differentiation of oligodendrocyte progenitors *in vitro* [26] to phosphorylate cytoskeletal proteins critical for differentiation and/or migration such as WAVE2 [28] and paxillin [27]. While we cannot rule out that the reduction in number of oligodendrocytes in our results is due to impaired differentiation, our observation of fewer Ki67+RIP+ cells suggest that the deficit in iCdk5 mice is due to reduced proliferation. However, Cdk5 may not directly control cell cycle [33] but rather may indirectly affect cell cycle and proliferation [72]; thus, our observation of reduced proliferation is most likely not directly downstream of Cdk5.

In addition to proliferation and differentiation, Cdk5 may also support postnatal gliogenesis by regulating glial migration [20,28,60]. Given the role of Cdk5 in neuronal migration [19,63], the normal pattern and/or timing of glial migration could also be disturbed in iCdk5 mice. Understanding possible glial migrational roles of Cdk5 in nestin-lineage progenitors would reveal common or divergent roles for this regulator in neurons vs. glial cells, similar to what has been found using *in vivo* [19,20,73] and *in vitro* [26–28] migrational assays. Future experiments examining the role of Cdk5 in early postnatal glial migration of nestin-expressing progenitors as well as the biochemical and molecular underpinnings of its role would therefore require separate, comprehensive study employing *in vivo*, *in vitro*, and *ex vivo* strategies [20,62,74–79]. It is also possible that in addition to impaired proliferation, differentiation/maturation, and migration, early deficits in the nestin-expressing progenitors may contribute to the iCdk5 phenotype. This is plausible, since Cdk5 can phosphorylate both GFAP and nestin, cytoskeletal proteins expressed in both astrocytes and neural progenitors [15,25,50,80].

In summary, our results demonstrate that nestin-expressing progenitors are capable of generating astrocytes and oligodendrocytes in different brain regions during a critical period of the first postnatal week in mice. Our results also show that Cdk5 is required for the proper early postnatal gliogenesis *in vivo*. As the number of cellular processes regulated by Cdk5 increases, and Cdk5 is implicated in neuron-glia interaction as a part of etiology of neurodegenerative diseases [24,33,81], these findings will help us better understand the role of Cdk5 in development of non-neuronal brain cells.

## Supporting Information

Figure S1 **Influence of Tamoxifen (Tam) administration to lactating mothers (TOM) and to mothers and pups (TOM-TOP) on Cre-mediated recombination and body weight.** In TOM experiments, only mothers received Tam (see Methods). In TOM-TOP experiments, Tam was administered orally to pups and their mothers (i.p.) once a day from P2-P4 or P7-P9. (**A**) The percent of nestin-YFP offspring from TOM vs. TOM-TOP litters that had evidence of recombination (e.g. YFP+ cells in any brain region) is 100% in TOM-TOP mice, but lower in TOM mice. Shown are data from Tam P2-P4 and kill P21. (**B**, **C**) TOM-TOP does not negatively influence normal progression of body weight in WT and iCdk5 littermates given Tam from either P2-P4 (**B**) or P7-P9 (**C**).
(TIFF)
