# Supplementary figures and images for: Early Postnatal In Vivo Gliogenesis From Nestin-Lineage Progenitors Requires Cdk5
Source: PLoS One. 2013 Aug 26;8(8):e72819. doi: 10.1371/journal.pone.0072819 (PMC3753242; doi:10.1371/journal.pone.0072819)

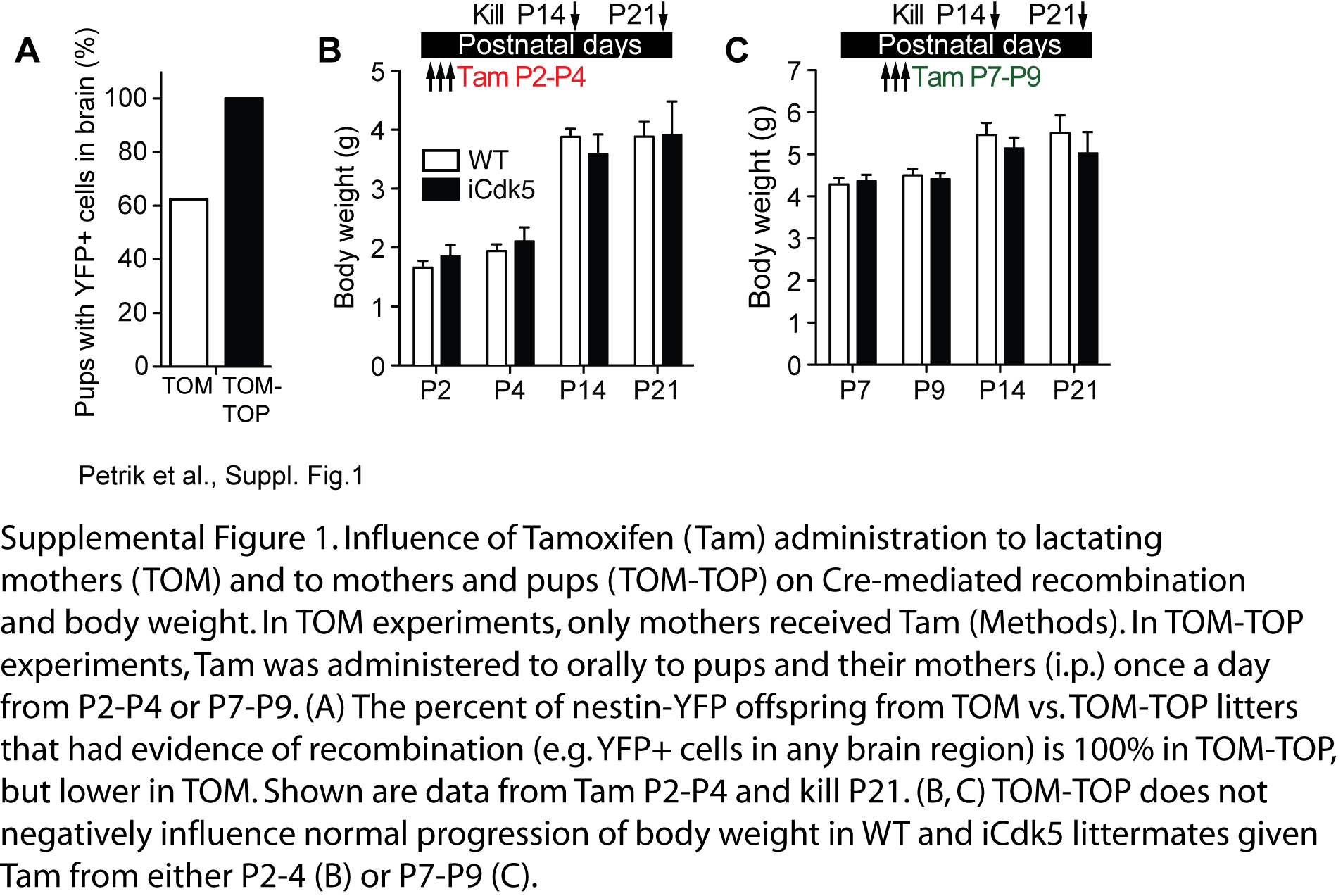

Supplement: Figure S1 — Influence of Tamoxifen (Tam) administration to lactating mothers (TOM) and to mothers and pups (TOM-TOP) on Cre-mediated recombination and body weight. In TOM experiments, only mothers received Tam (see Methods). In TOM-TOP experiments, Tam was administered orally to pups and their mothers (i.p.) once a day from P2-P4 or P7-P9. (A) The percent of nestin-YFP offspring from TOM vs. TOM-TOP litters that had evidence of recombination (e.g. YFP+ cells in any brain region) is 100% in TOM-TOP mice, but lower in TOM mice. Shown are data from Tam P2-P4 and kill P21. (B, C) TOM-TOP does not negatively influence normal progression of body weight in WT and iCdk5 littermates given Tam from either P2-P4 (B) or P7-P9 (C). (TIFF) [file pone.0072819.s001.tiff]
